# Supplementary material for: Enhancing, controlling, and sterilizing dengue immunity and the development of broadly protective responses
Source: medRxiv. 2026 Mar 27:2026.03.26.26349424. Preprint. [Version 1] doi: 10.64898/2026.03.26.26349424 (PMC13042151; doi:10.64898/2026.03.26.26349424)
Supplement: Supplement 1 [file media-1.pdf]

Supplementary Information for

Enhancing, controlling, and sterilizing dengue immunity and the development of broadly protective responses

This PDF file includes: Supplementary Figs. 1-3 and Tables 1-4

Other Supplementary Materials for this manuscript include: Supplementary Data Files 1-4

A

Reagent	Clone	Source	Catalog #	Dilution / Concentration
Golgi-Plug		BD Biosciences	555029	1:1000
Golgi-Stop		BD Biosciences	554724	1:1000
LIVE/DEAD Fixable Blue Dead Cell Stain		Thermo Fisher Scientific	L23105	1:50
Human BD Fc Block		BD Biosciences	564220	1:20
Brilliant Stain Buffer Plus		BD Biosciences	566385	1:10
CD40 anti-human	HB14	Miltenyi	130-094-133	0.5 μg/mL
Brilliant Violet 711 anti-human CD197 (CCR7)	G043H7	BioLegend	353228	1:160
BV605 anti-human CD69	FN50	BD Biosciences	562989	1:250
PE-Cy5 anti-human CD137	4B4-1	BioLegend	309808	1:500
BUV805 anti-human CD8	SK1	BD Biosciences	612889	1:200
Brilliant Violet 510 anti-human CD16*	3G8	BioLegend	302048	1:200
Brilliant Violet 510 anti-human CD14*	63D3	BioLegend	367124	1:200
Brilliant Violet 510 anti-human CD20*	2H7	BioLegend	302340	1:200
PE-Cy7 anti-human CD134 (OX40)	ACT35	BD Biosciences	563663	1:160
BUV395 anti-human CD3	UCHT1	BD Biosciences	563546	1:100
Brilliant Violet 570 anti-human CD45RA	HI100	BioLegend	304132	1:100
cFluor BYG750 anti-human CD4	SK3	Cytek Biosciences	SKU R7-20159	1:100
APC-efluor-780 anti-human CD154 (CD40 Ligand)	24-31	Thermo Fisher Scientific	47-1548-42	1:80
BB700 anti-human IFN gamma	B27	BD Biosciences	566394	1:80

*Antibodies make up the dump channel.

B

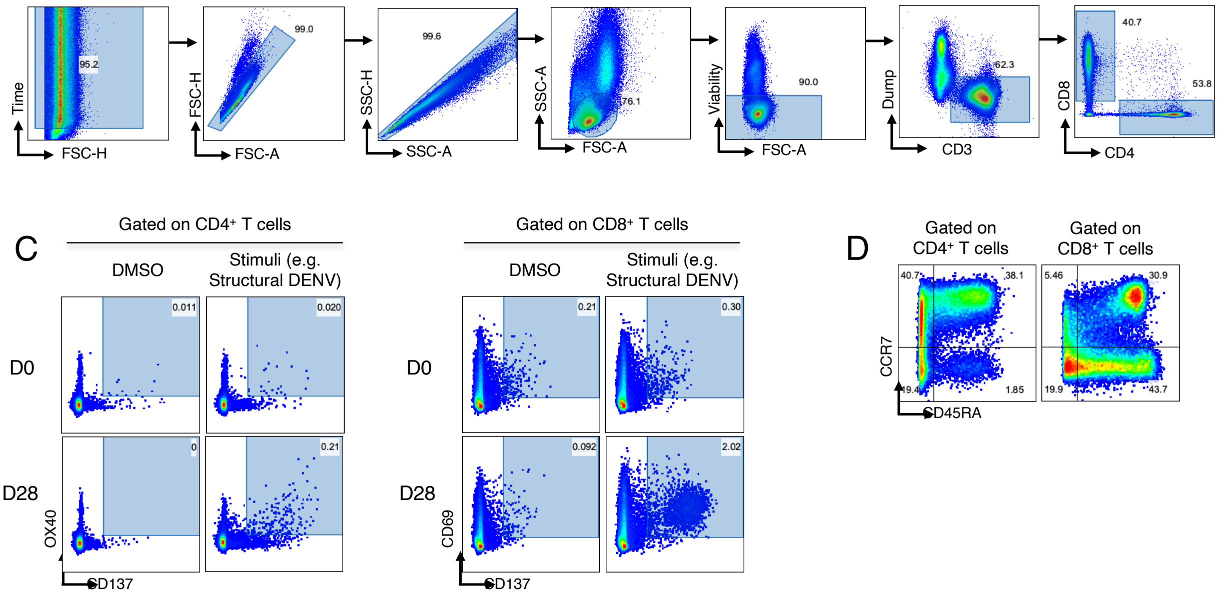

Supplementary Fig. 1. Representative gating strategy for T cell analysis. (**A**) Reagents. (**B**) Representative gating strategy to define viable CD4^{+} and CD8^{+} lymphocytes. (**C**) Representative gating strategy to define activated induced markers (AIM). (**D**) Representative gating strategy to define memory subsets.

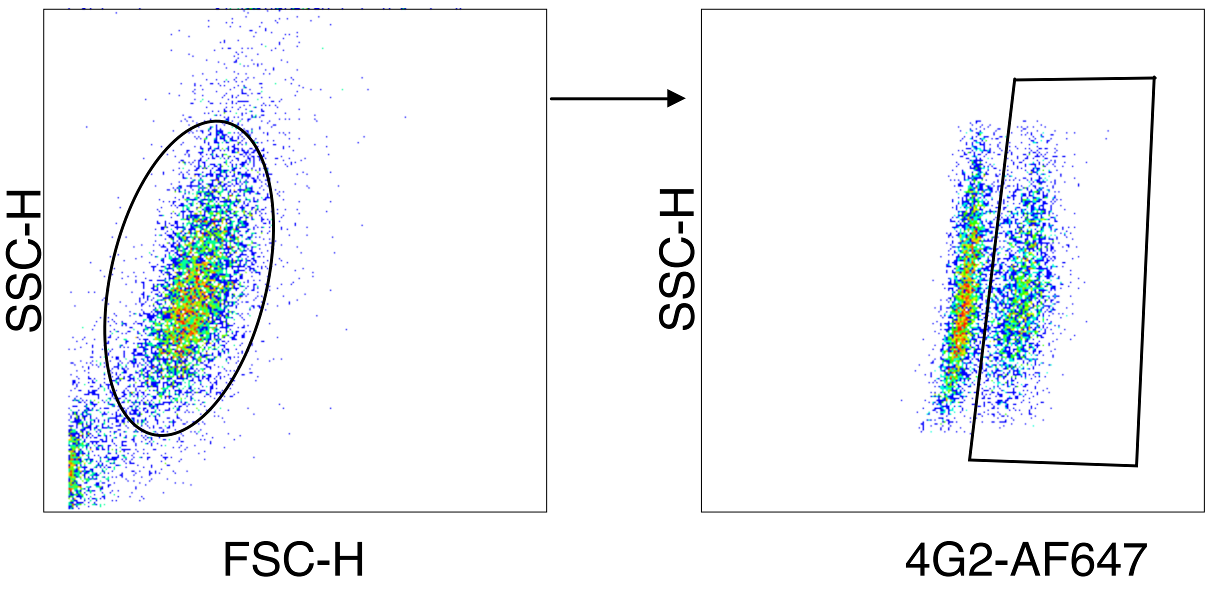

Supplementary Fig. 2. Representative gating strategy for ADE analysis. Gating scheme to define DENV-infected U937 cell lines expressing CD16.

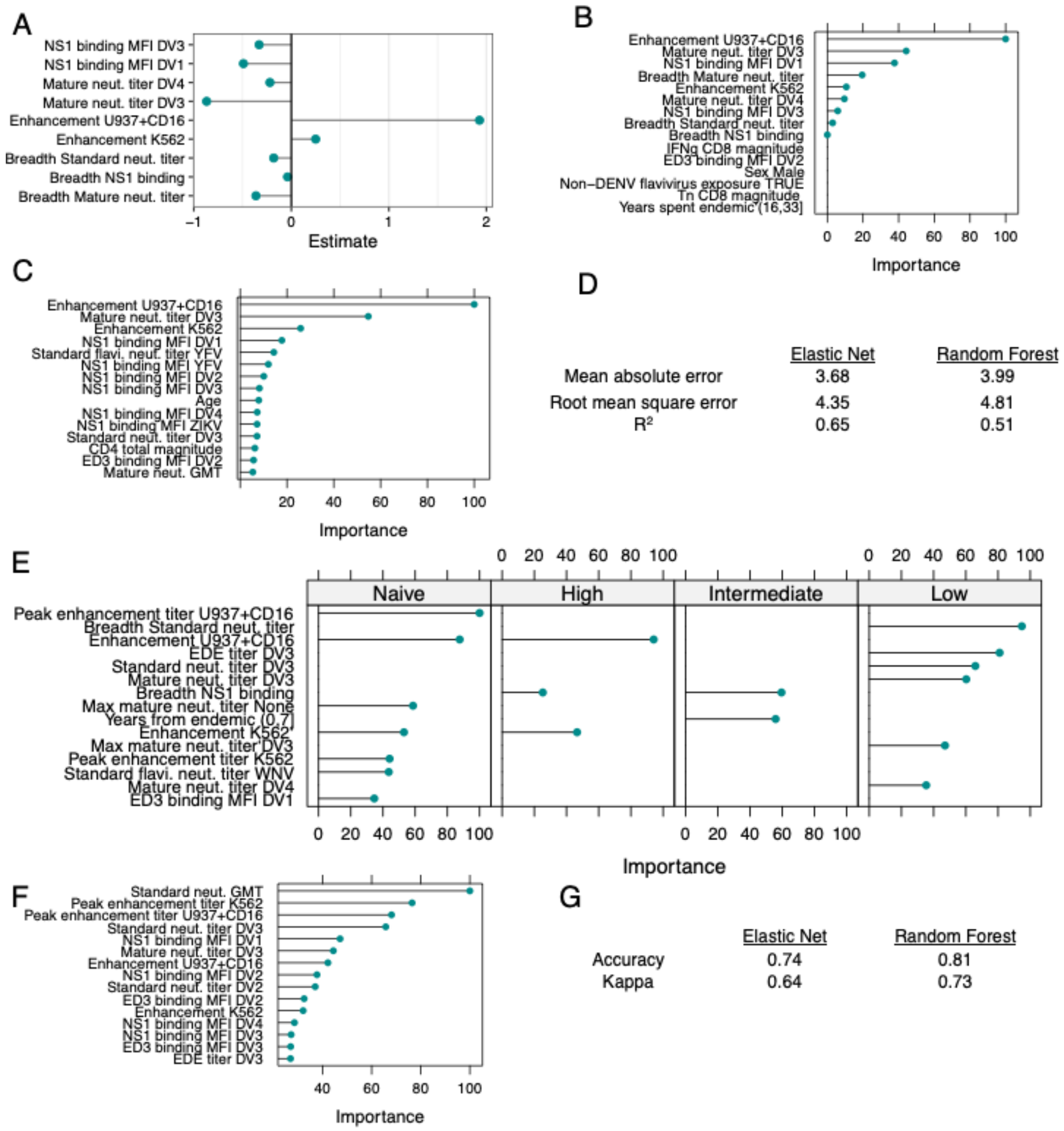

Supplementary Fig. 3. Immune measures identified as important predictors of viremia using machine learning methods. Coefficient effect sizes (**A**) and variable importance (**B**) for an elastic net model and variable importance for a random forest (**C**) model evaluating important immune and demographic predictors of AUC viremia. (**D**) Performance of each model was evaluated using cross-validation. Variable importance for elastic net (**E**) and random forest (**F**) multinomial models evaluating important immune and demographic predictors of each viremia

group. (**G**) Performance of each model in classifying groups was evaluated using cross-validation.

Number	Infection history group	DENV1	DENV2	DENV3	DENV4	GMT
1	Naive	5	5	5	5	5
2	Naive	5	5	5	5	5
3	Naive	5	5	5	5	5
4	Naive	5	5	5	5	5
5	Naive	5	5	5	5	5
6	Naive	5	5	5	5	5
7	Naive	5	5	5	5	5
8	Naive	5	5	5	5	5
9	Naive	5	5	5	5	5
10	Naive	5	5	5	5	5
11	Naive	5	5	5	5	5
12	Naive	5	5	5	5	5
13	Naive	5	5	5	5	5
14	Naive	5	5	5	5	5
15	Heterotypic	14	5	5	5	6
16	Heterotypic	18	5	5	5	7
17	Heterotypic	5	39	5	5	8
18	Heterotypic	5	71	5	5	10
19	Heterotypic	5	332	5	5	14

20	Heterotypic	5	375	5	5	15
21	Heterotypic	91	11	12	5	16
22	Heterotypic	10	642	17	5	27
23	Heterotypic	546	78	17	5	29
24	Heterotypic	5	4302	11	5	33
25	Heterotypic	5	371	28	33	36
26	Heterotypic	415	85	14	5	39
27	Heterotypic	11	507	27	24	43
28	Heterotypic	727	44	27	5	46
29	Heterotypic	24	429	21	24	48
30	Heterotypic	15	1302	43	39	76
31	Heterotypic	15	4287	29	21	79
32	Heterotypic	475	12812	39	592	614
33	Polytypic low	5	32	20	5	11
34	Polytypic low	5	42	20	5	12
35	Polytypic low	23	51	50	161	55
36	Polytypic	101	76	22	10	36
37	Polytypic	16	21	102	133	47
38	Polytypic	14	136	129	19	47
39	Polytypic	145	82	32	12	47
40	Polytypic	20	265	241	20	71
41	Polytypic	208	115	37	57	85

42	Polytypic	194	207	135	22	105
43	Polytypic	251	112	100	83	123
44	Polytypic	529	558	177	187	314
45	Polytypic	73	896	245	658	320

Supplementary Table 1. Day 0 neutralizing antibody titers for all participants.

Characteristic	Naive<br>N = 14[1]	Heterotypic<br>N = 18[1]	Polytypic<br>N = 13[1]	p-value[2]
Yellow fever vaccination				0.001
No	14 (100%)	7 (39%)	6 (46%)	
Not sure	0 (0%)	4 (22%)	1 (7.7%)	
Yes	0 (0%)	7 (39%)	6 (46%)	
Japanese encephalitis vaccination				0.2
No	14 (100%)	13 (72%)	11 (85%)	
Not sure	0 (0%)	4 (22%)	1 (7.7%)	
Yes	0 (0%)	1 (5.6%)	1 (7.7%)	
Tick-borne encephalitis vaccination				0.14
No	14 (100%)	14 (78%)	10 (77%)	
Not sure	0 (0%)	4 (22%)	2 (15%)	
Yes	0 (0%)	0 (0%)	1 (7.7%)	
Zika case	0 (0%)	1 (5.6%)	0 (0%)	>0.9
Dengue case	0 (0%)	12 (67%)	10 (77%)	<0.001
Lab-confirmed dengue case	0 (0%)	7 (39%)	6 (46%)	0.007
Years since dengue case	NA	13 (6, 17)	15 (9, 18)	0.6

Not applicable (n)	14	6	3	
Years since living in endemic area	5 (4, 12)	7 (4, 10)	7 (3, 15)	0.9
Not applicable (n)	9	1	2	
Total time living in endemic area	21 (8, 28)	11 (4, 26)	19 (5, 23)	>0.9
Not applicable (n)	10	2	4	
Contacts with dengue exposure	2 (14%)	4 (22%)	8 (62%)	0.022
Community took preventative measures against dengue	4 (29%)	12 (67%)	9 (69%)	0.049

[1]Median (Q1, Q3); n (%).

[2]Kruskal-Wallis rank sum test; Fisher's exact test.

Supplementary Table 2. History of flavivirus vaccination, infection, and exposure risk.

Adverse Event	Naïve (N=14)	Heterotypic (N=18)	Polytypic (N=13)	p-value[1]
Dengue-Like Rash	78.6	44.4	23.1	0.015
Feverish (subjective)	0	0	23.1	0.078
Diarrhea (>1 loose stool/day)	0	22.2	0	0.097
Myalgia	7.1	38.9	15.4	0.107
Respiratory rate >16 breaths per minute	21.4	27.8	0	0.117
Diastolic blood pressure >90 mmHg	14.3	0	23.1	0.124
Aspiration site bruising	21.4	22.2	0	0.182
Arthralgia	0	22.2	7.7	0.187
Headache	57.1	27.8	30.8	0.22
Glucose >99 (fasting) or >109 (random) mg/dL	50	22.2	38.5	0.27
Venipuncture site bruise	14.3	0	0	0.271
Hemoglobin <12.6 (male) or 11.1 (female) g/dL	21.4	44.4	23.1	0.33
Neutrophils <1,500 K/uL	21.4	44.4	23.1	0.33
Bruising	0	16.7	7.7	0.349
Fibrinogen >399 mg/dL	0	5.6	15.4	0.396
Acid reflux (esophageal)	0	11.1	0	0.456
Increased thirst	0	11.1	0	0.456
Injection site pain	0	11.1	0	0.456
Photophobia	0	11.1	0	0.456

Injection site tenderness	0	11.1	0	0.456
Potassium <3.7 mmol/L	28.6	11.1	23.1	0.458
Heart rate >100 beats per minute	14.3	11.1	0	0.461
Lymphocyte count <1,001K/uL	7.1	22.2	7.7	0.468
Malaise	7.1	22.2	7.7	0.468
Rash	7.1	22.2	7.7	0.468
Leukocyte count <3,501 K/mcL	21.4	38.9	23.1	0.528
Systolic blood pressure >140 mmHg	7.1	0	7.7	0.561
Hemoglobin drop >1.5 gm/dL from baseline	0	11.1	7.7	0.562
Retro-orbital pain	0	11.1	7.7	0.562
Liver enzymes >1.0x upper limit normal (ULN)	0	0	7.7	0.577
Hypersensitivity	0	0	7.7	0.577
Paresthesia	0	0	7.7	0.577
Polydipsia	0	0	7.7	0.577
BUN >22 mg/dL	0	0	7.7	0.577
C-reactive protein >5 mg/L	0	0	7.7	0.577
Insect bite	0	0	7.7	0.577
Shortness of breath	0	0	7.7	0.577
Sunburn-like rash	0	0	7.7	0.577
Tenderness	0	0	7.7	0.577
Vasovagal reaction	7.1	11.1	0	0.605

Creatinine kinase >1.24x ULN	7.1	0	0	0.622
Dry skin	7.1	0	0	0.622
Dysuria	7.1	0	0	0.622
Hoarseness	7.1	0	0	0.622
Cold sores	7.1	0	0	0.622
Cough	7.1	0	0	0.622
Joint pain	7.1	0	0	0.622
Nasal congestion	7.1	0	0	0.622
Presyncope	7.1	0	0	0.622
Venipuncture site pain	7.1	0	0	0.622
Fatigue	57.1	50	38.5	0.639
Chills	7.1	16.7	7.7	0.73
AST >1x ULN	14.3	5.6	7.7	0.73
Sore throat	14.3	5.6	7.7	0.73
Aspiration site pain	21.4	33.3	23.1	0.748
Injection site redness	7.1	5.6	0	0.777
ALT >1x ULN	7.1	5.6	0	0.777
Viral infection NOS	7.1	5.6	0	0.777
Abdominal cramps	0	5.6	0	0.799
Aspiration site swelling	0	5.6	0	0.799
Constipation	0	5.6	0	0.799
Decreased appetite	0	5.6	0	0.799
Difficulty sleeping	0	5.6	0	0.799

Dizziness	0	5.6	0	0.799
Drenching sweats	0	5.6	0	0.799
Eye pain	0	5.6	0	0.799
Fall	0	5.6	0	0.799
Herpes simplex type I	0	5.6	0	0.799
Potassium >5 mEq/L	0	5.6	0	0.799
Systolic blood pressure <90 mmHg	0	5.6	0	0.799
Injection site erythema	0	5.6	0	0.799
Low back pain	0	5.6	0	0.799
Night sweats	0	5.6	0	0.799
PTT >1x ULN	0	5.6	0	0.799
Periorbital edema	0	5.6	0	0.799
Pneumonia	0	5.6	0	0.799
Sinus congestion	0	5.6	0	0.799
Urinary frequency	0	5.6	0	0.799
Urinary tract infection	0	5.6	0	0.799
Glucose <70 mg/dL	14.3	16.7	7.7	0.818
Lymphadenopathy	14.3	11.1	7.7	0.895
Heart rate <55 beats per minute	7.1	11.1	7.7	0.955
Sodium <135 mEq/L	7.1	5.6	7.7	0.958
Nausea	14.3	16.7	15.4	0.991

[1]p-value for likelihood ratio test, overall differences among groups.

Supplementary Table 3. Frequency (%) of all adverse events through day 28 across immune groups based on day 0 neutralizing antibody titers.

Adverse Event	Naïve (N=14)	Heterotypic (N=18)	Polytypic (N=13)	p-value[1]
Dengue-Like Rash	78.6	44.4	23.1	0.015
Feverish (subjective)	0	0	23.1	0.078
Myalgia	7.1	33.3	15.4	0.209
Diarrhea (>1 loose stool/day)	0	16.7	0	0.221
Diastolic blood pressure >90 mmHg	14.3	0	0	0.271
Headache	50	27.8	23.1	0.306
Neutrophils <1,500 K/uL	21.4	44.4	23.1	0.33
Leukocyte count <3,501 K/mcL	21.4	38.9	15.4	0.348
Arthralgia	0	16.7	7.7	0.349
Increased thirst	0	11.1	0	0.456
Injection site pain	0	11.1	0	0.456
Photophobia	0	11.1	0	0.456
Injection site tenderness	0	11.1	0	0.456
Lymphocyte count <1,001K/uL	7.1	22.2	7.7	0.468
Malaise	7.1	22.2	7.7	0.468
Retro-orbital pain	0	11.1	7.7	0.562

Liver enzymes >1.0x upper limit normal (ULN)	0	0	7.7	0.577
Hypersensitivity	0	0	7.7	0.577
Paresthesia	0	0	7.7	0.577
Polydipsia	0	0	7.7	0.577
C-reactive protein >5 mg/L	0	0	7.7	0.577
Shortness of breath	0	0	7.7	0.577
Tenderness	0	0	7.7	0.577
Rash	7.1	11.1	0	0.605
Joint pain	7.1	0	0	0.622
Systolic blood pressure >140 mmHg	7.1	0	0	0.622
Vasovagal reaction	7.1	0	0	0.622
Fatigue	57.1	50	38.5	0.639
Bruising	0	5.6	7.7	0.721
Chills	7.1	16.7	7.7	0.73
Lymphadenopathy	14.3	5.6	7.7	0.73
Injection site redness	7.1	5.6	0	0.777
ALT >1x ULN	0	5.6	0	0.799
Aspiration site swelling	0	5.6	0	0.799
Decreased appetite	0	5.6	0	0.799
Difficulty sleeping	0	5.6	0	0.799
Dizziness	0	5.6	0	0.799

Drenching sweats	0	5.6	0	0.799
Injection site erythema	0	5.6	0	0.799
Low back pain	0	5.6	0	0.799
Night sweats	0	5.6	0	0.799
PTT >1x ULN	0	5.6	0	0.799
Periorbital edema	0	5.6	0	0.799
Pruritus	0	5.6	0	0.799
Heart rate >100 beats per minute	0	5.6	0	0.799
Nausea	14.3	11.1	15.4	0.925
AST >1x ULN	7.1	5.6	7.7	0.958

[1]p-value for likelihood ratio test, overall differences among groups

Supplementary Table 4. Frequency (%) of vaccine-associated adverse events across immune groups based on day 0 neutralizing antibody titers.
